# RNAseq Analysis Highlights Specific Transcriptome Signatures of Yeast and Mycelial Growth Phases in the Dutch Elm Disease Fungus *Ophiostoma novo-ulmi*

**DOI:** 10.1534/g3.115.021022

**Published:** 2015-09-17

**Authors:** Martha Nigg, Jérôme Laroche, Christian R. Landry, Louis Bernier

**Affiliations:** * Institut de Biologie Intégrative et des Systèmes (IBIS), Université Laval, Québec, G1V 0A6, Canada; † Centre d’Étude de la Forêt (CEF) and Département des sciences du bois et de la forêt, Université Laval, Québec, G1V 0A6, Canada; ‡ Plate-forme de bio-informatique, Institut de Biologie Intégrative et des Systèmes (IBIS), Université Laval, Québec, G1V 0A6, Canada; § Département de biologie, Université Laval, Québec, G1V 0A6, Canada

**Keywords:** *Ophiostoma novo-ulmi*, fungal dimorphism, transcription profiling, *Histoplasma capsulatum*, *Candida albicans*

## Abstract

Fungal dimorphism is a complex trait and our understanding of the ability of fungi to display different growth morphologies is limited to a small number of model species. Here we study a highly aggressive dimorphic fungus, the ascomycete *Ophiostoma novo-ulmi*, which is a model in plant pathology and the causal agent of Dutch elm disease. The two growth phases that this fungus displays, *i.e.*, a yeast phase and mycelial phase, are thought to be involved in key steps of disease development. We used RNAseq to investigate the genome-wide gene expression profiles that are associated with yeast and mycelial growth phases *in vitro*. Our results show a clear molecular distinction between yeast and mycelial phase gene expression profiles. Almost 12% of the gene content is differentially expressed between the two phases, which reveals specific functions related to each growth phase. We compared *O. novo-ulmi* transcriptome profiles with those of two model dimorphic fungi, *Candida albicans* and *Histoplasma capsulatum*. Few orthologs showed similar expression regulation between the two growth phases, which suggests that, globally, the genes associated with these two life forms are poorly conserved. This poor conservation underscores the importance of developing specific tools for emerging model species that are distantly related to the classical ones. Taken together, our results provide insights into transcriptome regulation and molecular specificity in *O. novo-ulmi* and offer a new perspective for understanding fungal dimorphism.

Morphological dimorphism allows several human and plant pathogenic fungi to live either in a unicellular yeast phase or in a pluricellular mycelium stage. This characteristic provides an advantage to pathogenic fungi that colonize various environments during their lifecycle (Nemecek *et al.* 2006). Our understanding of the diversity of molecular processes that are involved in this morphological plasticity is rather limited due to the small number of species that have been studied, and which consist mostly of human pathogenic fungi for which dimorphism is thermally regulated. More investigations are needed to overcome this limitation and to cover a wider spectrum of the fungal kingdom. Here we develop an additional experimental system to study genes and pathways underlying dimorphism using the ascomycete *Ophiostoma novo-ulmi*.

This dimorphic fungus is a vascular plant pathogen that is responsible for the current pandemic of Dutch elm disease (DED), which has been killing elm tree (*Ulmus* sp.) populations in North America and Europe since the 1940s. *Ophiostoma novo-ulmi* is closely related to *O. ulmi*, which caused the first DED pandemic at the beginning of the 20^th^ century (Brasier 1991). *O. novo-ulmi* is phylogenetically closely related to the dimorphic Ophiostomatoid human pathogen *Sporothrix schenckii* and to *Neurospora crassa*, a model fungus from the class Sordariomycetes (Berbee and Taylor 1992; Hintz 1999) (Figure 1).

**Figure 1:**
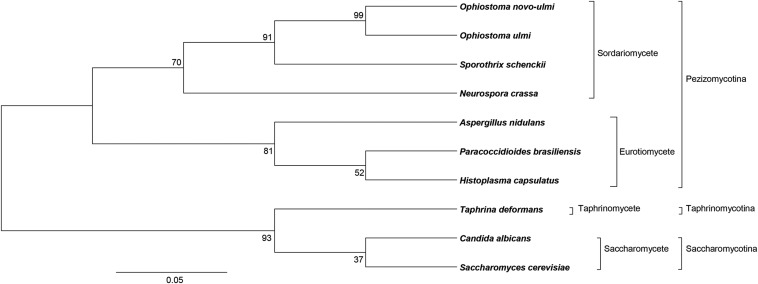
Phylogeny of ascomycete fungi based on concatenated Internal Transcribed Spacer 1 sequences (ITS1). Except for *Neurospora crassa*, all of these fungi are dimorphic. ITS sequences were downloaded from the NCBI database and exported to BioEdit 7.2.5 software (Hall 1999) to align and concatenate the sequences (see strain or isolate names and references in Table S1). A maximum-likelihood tree was constructed using Mega6 with 1000 bootstrap (Tamura *et al.* 2013). Bootstrap support is shown on each node. Sordariomycete, Eurotiomycete, Taphrinomycete, and Saccharomycete are classes. Pezizomycotina, Taphrinomycotina and Saccharomycotina are subdivisions of the ascomycetes.

The yeast-to-mycelium transition of *O. novo-ulmi* occurs at the haploid stage (Pereira *et al.* 2000) and is not dependent on sexual reproduction or temperature. Both phases seem to be required to insure the complete infection of elm trees (Miller and Elgersma 1976). The nuclear genome of *O. novo-ulmi* was recently sequenced (Forgetta *et al.* 2013; Bernier *et al.* 2015) and contains approximately 8640 coding genes, of which approximately 25% remain unannotated (Comeau *et al.* 2015). Knowing which of these genes are expressed specifically in the yeast and mycelium phases remains undetermined because data for *O. novo-ulmi* consist largely of expressed sequence tags (ESTs) that are associated with the yeast phase (Hintz *et al.* 2011) and the production of fruiting bodies (Jacobi *et al.* 2010). However, identifying the genes that are essential for maintaining yeast and mycelium growth phases is important to better understand diseases that are caused by dimorphic fungi and, eventually, to manipulate the switch between these two morphs.

Here we characterize and quantify the transcriptome of yeast and mycelial forms of *O. novo-ulmi* in stable (*i.e.*, not in transition) growth stages using RNAseq. To assess the proportion of conserved expression patterns between *O. novo-ulmi* and well-established model species, we compare our data with those from two model species, *Candida albicans* and *Histoplasma capsulatum* (both dimorphic human pathogens), for which genomes and transcriptomes are available. Overall, our results provide new insights into the transcriptomic profiles that differentiate growth phases in *O. novo-ulmi* and establish a new model for the study of dimorphism in a phytopathogenic fungus.

## Materials and Methods

### Strains and samples

Yeast and mycelial forms of *O. novo-ulmi* strain H327 (Centre d’Étude de la Forêt, fungal collection) were grown on *Ophiostoma* complete medium (OCM) with 1.15 g L^−1^ proline as the nitrogen source in incubators that were set at 22° (Bernier and Hubbes 1990). Three different life stages (treatments) were analyzed: (1) yeast: 5-d-old liquid cultures of yeast cells in agitated flasks on an orbital shaker (Infors HT Ecotron) at 150 rpm (initial concentration of spores: 1 × 10^5^ mL^−1^); (2) liquid mycelium: 5-d-old liquid culture of mycelium without shaking (initial concentration of spores: 2.5 × 10^4^ mL^−1^); and (3) solid mycelium: 5-d-old solid culture (complete medium with 20 g L^−1^ agar in Petri plates) of mycelium (1.5 × 10^6^ spores spread on sterile cellophane membrane). In liquid medium, different spore concentrations were used for yeast and mycelial growth to prevent hyphal formation in the shaken medium by quorum sensing (Berrocal *et al.* 2012; M.E. Wedge and L. Bernier, unpublished results). Each treatment included three biological replicates (Supporting Information, Figure S1).

### RNA extraction, cDNA library production, and RNA sequencing

Samples were harvested by centrifugation (5 min, 4700 g, 4°) for liquid cultures or by collecting mycelium directly on the membrane for solid cultures. Samples were then stored at −80°. Total RNA was extracted using the RNeasy Mini Kit (QIAGEN, yeast protocol) and quality control was assured using a spectrophotometer (NanoDrop ND-1000, Thermo Scientific) and a bioanalyzer (BioAnalyzer RNA 6000 Nano Kit, Agilent Technologies). Complementary DNA (cDNA) libraries were synthesized using the TruSeq RNA Sample Preparation Kit v2 (Illumina) with 1 μg of starting material per sample at the Plateforme d’Analyses Génomiques (IBIS/Université Laval). Libraries were sequenced on an Illumina HiSequation 2500 (v.1.9 single-end, 100 bp) platform at McGill University and the Genome Québec Innovation Centre. The nine bar-coded samples were multiplexed with three other samples (unpublished data) in the same sequencing lane. Two of the nine samples (one from yeast phase and one from mycelium phase grown on solid medium) were used by Comeau *et al.* (2015) to improve genome annotation. All nine RNAseq samples are available under the NCBI BioProject (PRJNA260920) on Genbank: the three replicates of yeast growth phase (accession numbers: SRR1574322, SRR2140671-2); the three replicates of mycelial phase grown on solid medium (accession numbers: SRR1574324, SRR2140674-5); and the three replicates of mycelial phase grown in liquid medium (accession numbers: SRR2140676-8).

### Data preprocessing

Filtering and quality controls were applied following the procedure described by Comeau *et al.* (2015). Adaptors and poly-A/T tails (>10 nt) were trimmed off with a combination of Trim Galore! (www.bioinformatics.babraham.ac.uk/projects/trim_galore/) and Prinseq v0.20.4 (Schmieder and Edwards 2011). Sequences containing more than 20% Ns or shorter than 20 nt in length were removed. We also discarded duplicate reads from our data (duplicates can be removed if sequences are exactly the same, without allowing any mismatches, because they are considered PCR duplicates). Because the percentage of reads that were eliminated was homogenous among conditions (Figure S2A, Table S2), we assumed that this would not bias the analysis. The remaining number of reads that were analyzed fell between 3.7 and 5.7 million per sample.

### Data analysis

Filtered reads were mapped onto the *O. novo-ulmi* H327 genome (Forgetta *et al.* 2013) with TopHat2 (v.2.0.10) using default parameters (Kim *et al.* 2013). Reads were also mapped in parallel on exon sequences to assess the proportion that had mapped onto noncoding sequences. All further analyses were performed using R (v3.0.1; R Core Team 2015). Read counts for each of the nine samples were extracted and used for differential expression analyzes using the *EdgeR* package in *Bioconductor 3.0* (Robinson *et al.* 2009). For downstream analyses, we considered only the genes with 25 reads or greater in at least three samples. Library sizes (*i.e.*, number of mapped reads) were corrected using a method based on Trimmed Mean of M-values (TMM) (Robinson and Oshlack 2010; Dillies *et al.* 2013). A multidimensional scaling (MDS) plot was used for the visualization of the variability among samples (function implemented in *EdgeR*). In the MDS plot, distances correspond to the average of the largest absolute log-fold changes (logFC) between each pair of RNA samples (Robinson *et al.* 2009). The nine samples were clustered using the *k*-means method (Hartigan and Wong 1979) and the plot was produced using the R package *ggplot2* (Wickham 2009).

Differential expression levels (relative RNA counts) between yeast and each of the two mycelial conditions were considered significantly different with a false discovery rate (FDR) (Benjamini and Hochberg 1995) at a threshold of 1%. Gene Ontology (GO) terms were attributed using BLAST2GO (www.blast2go.com; 3162 *O. novo-ulmi* H327 genes do not have a GO term) (Comeau *et al.* 2015). Enrichment was computed using *GOStats* and *Goseq* packages in *Bioconductor 3.0* (Young *et al.* 2010). Enriched GO terms with *P*-values (uncorrected) ≤1% and a minimum of four genes per category were retained and trimmed off with GO Trimming to reduce term overlap (80% soft trim threshold) (Jantzen *et al.* 2011).

### Gene orthology

Orthologous genes for species pairs (see species names below) were identified by reciprocal best blast hits (RBH) using *tblastx* (Altschul *et al.* 1990). Exon sequences of the two species were compared using local databases and the RBH was conducted using the following parameters: e-value = 1 × 10^−3^ and word size = 5. The parameters were validated by calculating the number of orthologous genes between *O. novo-ulmi* or *O. ulmi* (gene sequences downloaded on 4 April 2015, www.moseslab.csb.utoronto.ca/o.ulmi/, “nuc-seq-all-genes” named sequences) and *Neurospora crassa* (sequences retrieved on 31 March 2015, www.broadinstitute.org/annotation/genome/neurospora/, “or74a_12” named sequences). We also calculated the number of orthologous genes between *Ophiostoma* sp. and *Saccharomyces cerevisiae* strain S288C (www.yeastgenome.org/, “R63-1-120100105” named sequences) (Table S3). We compared our results with those obtained by Khoshraftar *et al.* (2013). Despite using methods that differed from those of Khoshraftar *et al.* (2013), our estimates were similar to the values that these authors obtained. Thus, we assume that our workflow is successful in identifying orthologous genes. The limited variation that was seen between numbers of orthologs found can be explained by the different versions of the databases used. The RBH method was used to identify orthologous genes between *O. novo-ulmi* and the reference strains of *Histoplasma capsulatum* (strain G186) and *Candida albicans* (strain SC5314). For *H. capsulatum*, *de novo* transcriptome assembly data (Edwards *et al.* 2013) were kindly provided by Dr. C. Rappleye (Ohio State University, Columbus, OH) and used for constructing the local Blast database. For *Candida albicans*, exon sequences were downloaded on 23 February 2015 (www.candidagenome.org/). The RBH method was also used to assess the number of orthologous genes between *O. novo-ulmi* and *Sporothrix schenckii* strain ATCC58251 (transcript sequences downloaded on 3 July 2015) (Cuomo *et al.* 2014). The total number of genes present in each species that was tested was retrieved from databases on 6 July 2015 for *S. schenckii* and on 8 May 2015 for all of the other species.

### Comparison with *Histoplasma capsulatum* and *Candida albicans* RNAseq data

Paired-end RNAseq data from *H. capsulatum* were downloaded from NCBI [www.ncbi.nlm.nih.gov/sra, accession numbers: SRX332751 (G186 mycelia, two files) and SRX332607 (G186 yeasts, two files)]. SRA files were converted into fastq format with the SRA toolkit (www.ncbi.nlm.nih.gov/Traces/sra/). Fastq files with both ends concatenated were split into two files (one for each end, forward and reverse) with Fastq-splitter (www.kirill-kryukov.com/study/tools/fastq-splitter/). Split files were cleaned using Prinseq v0.20.4 (Schmieder and Edwards 2011) and 156 nt long reads were trimmed to the first 70 nt (thereby avoiding the large proportion of “N” in the second half of reads). We analyzed differential gene expression following the method described above for *Ophiostoma* samples. The hypergeometric test was used to infer the probability of gene expression regulation conservation between two compared species. The same procedure was applied to *C. albicans* strain SC5314 data. Transcriptome data for *C. albicans* (Bruno *et al.* 2010) were downloaded from the NCBI SRA database (www.ncbi.nlm.nih.gov/sra, accession numbers: SRR064145-8). We selected samples corresponding to yeast form (accession numbers: SRR064145 and SRR064146) and hyphal growth (accession numbers: SRR064147 and SRR064148).

### Data availability

Strain H327 of *Ophiostoma novo-ulmi* is available upon request. Sequence data are available at Genbank and the accession numbers are listed at the end of the subsection “RNA extraction, cDNA library production and RNA sequencing”. Details for supplemental figures and tables are listed in the Supporting Information.

## Results and Discussion

*O. novo-ulmi* is a model species in terms of its interaction with the host tree, its epidemiology, and its high virulence. This fungus has been studied for many years with respect to its involvement in Dutch elm disease (Bernier *et al.* 2015). As is the case with many other dimorphic fungi, this pathogen is capable of living either in a yeast phase or in a mycelial phase, depending on environmental conditions. Even though the two growth phases can easily be obtained and controlled *in vitro* (Naruzawa and Bernier 2014), only a few studies have focused on the molecular landscape of this dimorphism, and only on a limited number of genes (Richards 1994; Pereira *et al.* 2000). Our study aimed at determining the gene expression profiles of different morphs of *O. novo-ulmi* to identify expression signatures that distinguish the yeast and mycelial phases. To do so, we sequenced the transcriptome of both phases of *O. novo-ulmi* in stable growth conditions (not in transition) and focused on the regulation of gene expression in yeast and mycelium.

### RNAseq data statistics

All samples (three treatment conditions, each with three replicates) produced approximately 13–15 million raw reads each (Figure S2A). The cleaned and filtered data represent a depth of coverage of 28× for exons and 13× for the whole genome. A total of 1.75% of reads is identified as mapping to noncoding regions, which represent noncoding RNA, introns, or untranslated regions (UTRs) of protein-coding genes.

Under each condition, one or more reads is mapped to at least 95% of the predicted genes in the *O. novo-ulmi* H327 genome. The distribution of the number of reads per gene is homogenous across conditions, suggesting that the transcriptomes of the two morphs have similar complexities (Figure S2B). Eight hundred ninety-nine predicted genes (Comeau *et al.* 2015) were not detected or were discarded from our dataset after the EdgeR filtration steps. Of these, 470 are predicted to encode as yet uncharacterized proteins. Among the remaining 429 genes, some are clearly condition-specific, such as mating-type genes *MAT1-1-1*, *MAT1-1-2*, and *MAT1-1-3*, and the pheromone mating α-factor receptor encoding genes *STE2* and *STE3*. These genes are related to mating conditions (Jacobi *et al.* 2010) that are distinct from the conditions that were examined here. Genes coding for salicylate hydroxylases (*n* = 7) and tannases (*n* = 4) were also absent. These enzymes are involved in plant metabolite degradation (Gaffney *et al.* 1993; García-Conesa *et al.* 2001) and, thus, may not be expressed in the set of conditions that we investigated. For further analyses, we consider the 7741 genes that are detected by EdgeR as being expressed (Table S4).

### Expression data visualization and reproducibility

**Figure 2:**
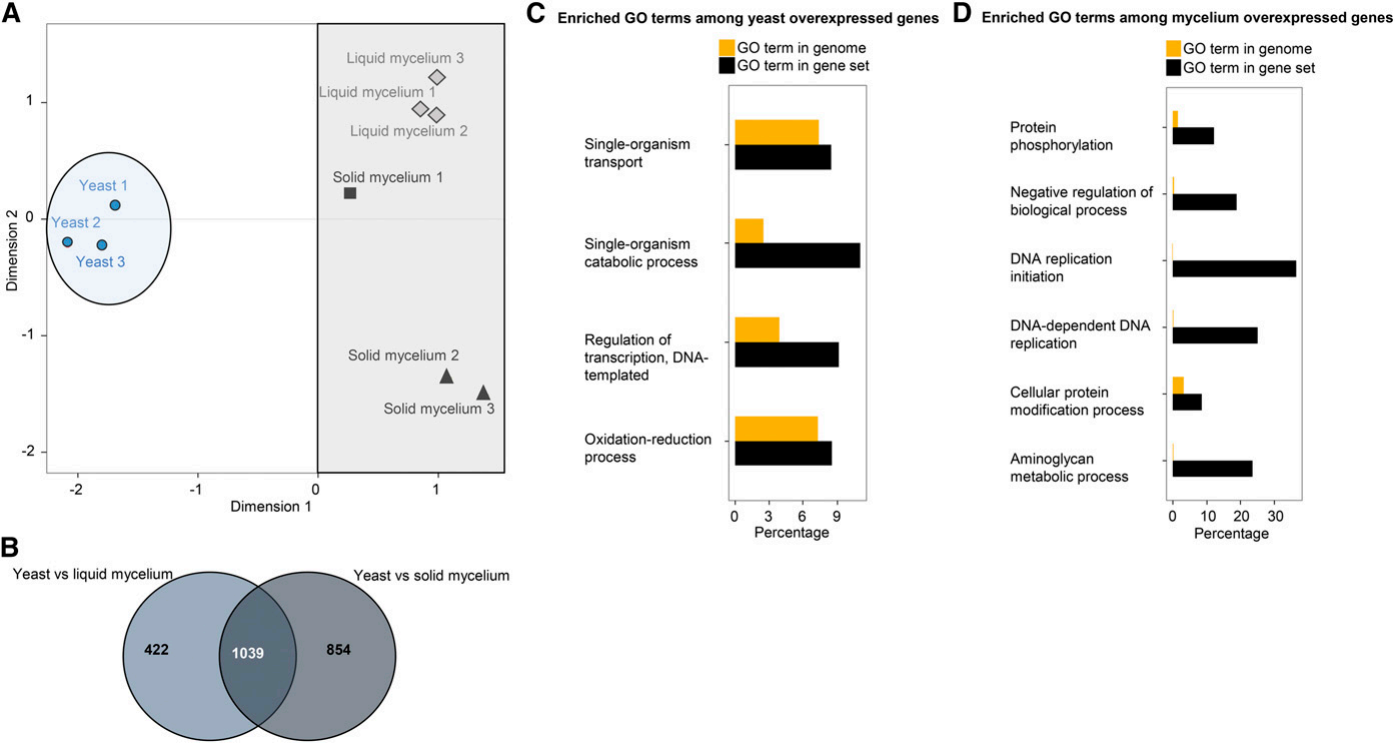
Sample variability, differential gene expression, and Gene Ontology (GO) Enrichment analysis between yeast and mycelium samples of *Ophiostoma novo-ulmi*. (A) Multi-dimensional scaling plot of the nine RNA samples sequenced from yeast phase, mycelium grown in liquid medium (liquid mycelium), and mycelium grown on Petri plates (solid mycelium) inoculated with *Ophiostoma novo-ulmi*. Dot shapes indicate results of cluster analysis by *k*-means test; dot colors, growth conditions; blue circle, yeast phase; grey rectangle, all the mycelium samples. (B) Venn diagram of the number of differentially expressed genes (FDR ≤1%), depending on the comparison being made: yeast *vs.* mycelium grown in liquid medium (1460 differentially expressed genes in total, light blue); yeast *vs.* mycelium grown on Petri dishes (1852 differentially expressed genes in total, grey). Number of differentially expressed genes that are shared between the comparisons: 1039. Enriched GO terms (biological processes) in *Ophiostoma novo-ulmi* for (C) 397 annotated overexpressed genes in yeast; (D) 334 annotated overexpressed genes grown in the two mycelial conditions (liquid medium *vs.* Petri plates). Yellow bars indicate percentage of the whole genome related to a GO term. Black bars indicate percentage of total genes in a GO term that are present in the overexpressed set of genes. *P* ≤ 0.01 (Fisher’s exact test implemented in GOseq).

MDS analysis shows the variability and grouping among samples (Figure 2A). The three yeast biological replicates cluster together, as do the three replicates of mycelium grown in static liquid conditions. Mycelium samples that were grown on solid medium exhibit greater variability than other treatments. The MDS plot shows clear separation between yeast samples and all mycelial samples along the first axis, which indicates that the largest fraction of variation measured in terms of gene expression is due to dimorphism. The second dimension discriminates the mycelium according to the medium that was used (liquid or solid), suggesting that the growth medium largely affects the transcriptome. Yeast samples are closer to the mycelium phase when grown in liquid medium than to mycelia that were grown in Petri dishes. Thus, some genes are differentially regulated according to whether the fungus is in liquid or solid medium.

### Phase-regulated gene expression

Gene expression levels of yeast samples were compared to each of the two mycelial conditions in pairwise comparisons (Figure 2B). This test allows the identification of genes that are systematically differentially expressed between yeast and mycelium samples and that could explain the separation of the two lifecycle stages along the first dimension of the MDS plot. A set of 1040 genes was differentially expressed in both comparisons [yeast *vs.* liquid mycelium; yeast *vs.* solid mycelium; log_2_ of fold change (logFC) from 0.75 to 8.5, FDR ≤1%]. For the majority (*n* = 1039), the pattern of overexpression or underexpression is consistent in both comparisons (Figure 2B). This number represents almost 12% of the predicted nuclear gene content of *O. novo-ulmi*. Among these 1039 genes, 538 are overexpressed in yeast samples and 501 are overexpressed in mycelium samples (Table S5 and Table S6). For both comparisons, the set of differentially expressed genes that do not overlap represents genes with expression profiles that depend on the growth conditions (liquid *vs.* solid medium). This could explain the differences seen in the second dimension of the MDS plot.

#### Single-cell lifestyle:

In the list of the 538 genes that were overexpressed in the yeast condition, 141 are annotated as “putative uncharacterized protein.”

Four biological processes stood out following GO terms enrichment analysis for the remaining 397 genes (Figure 2C). There are less than 60 genes in each of the four terms (Table S5). Among the latter, the term *regulation of transcription*, *DNA-templated* contains nine of the 15 transcription factors overexpressed in the yeast condition (Table S5). There are 159 genes annotated as transcription factors in the genome (Comeau *et al.* 2015). In the set of overexpressed genes in the yeast growth phase, we found C6 zinc finger transcription factors (*n* = 4) and Bzip transcription factors (*n* = 4), two of which are AP-1-like transcription factors (OphioH327g0401 and OphioH327g0658). In fungi, AP-1-like transcription factors are known to be involved in several processes such as the response to oxidative stress (Toone *et al.* 2001).

Another term, *single-organism catabolic process*, which represents less than 3% of the whole genome, contains enzymes such as dehydrogenases (*n* = 14), oxidoreductases (*n* = 7), or oxidases (*n* = 5) (Table S5). The role of the enzymes in the yeast growth phase is not yet understood. However, in this term, we found several genes encoding catalases (OphioH327g1152, OphioH327g3052, and OphioH327g6217), which are enzymes involved in antioxidant defenses. These gene functions have already been associated with a yeast-specific expression in *H. capsulatum* (Inglis *et al.* 2013). This suggests a conserved expression pattern for these genes. It has also been shown in *Paracoccidioides brasiliensis* and *Penicillium marneffei*, two dimorphic human pathogens, that gene expression and catalase activity are highly regulated with respect to the growth phase. For some of the catalases that have been described, both species show an increase in yeast phase (Pongpom *et al.* 2005; Xi *et al.* 2007; Chagas *et al.* 2008). Moreover, OphioH327g6217 is orthologous to *CAT1* of *Candida albicans* and *cat-4* of *Neurospora crassa*. In *C. albicans*, CAT1 is involved in hyphal differentiation in response to hydrogen peroxide and mutants of *CAT1* display abnormal hyphal growth (Nasution *et al.* 2008). Interestingly, *CAT1* expression is regulated by an AP-1-like transcription factor, *CAP1* (Alarco and Raymond 1999). The function and regulation of the *O. novo-ulmi CAT1* ortholog, however, are not known but could be conserved, and this gene could be involved in hyphal differentiation.

Although the OCM growth medium contained no urea, we found a gene encoding a urease, OphioH327g2055, which was overexpressed in the yeast phase (average logFC 1). In *P. brasiliensis*, a gene annotated as encoding the same enzyme is also preferentially expressed in the yeast phase (Felipe *et al.* 2005). An interesting aspect of urease is that this enzyme seems to have a conserved role across fungal and bacterial human infection as a general virulence factor for human pathogens (Rutherford 2014). Ureases particularly are required in one dimorphic and one saprophytic yeast fungi: *Cryptococcus neoformans* (Cox *et al.* 2000) and *Coccidioides posadasii* (Mirbod-Donovan *et al.* 2006), respectively. Our results may suggest that the role of this enzyme in fungal pathogenesis is conserved in *O. novo-ulmi*.

#### Filamentation signatures:

Of the 501 genes that were overexpressed in the mycelium condition, 167 are genes coding for uncharacterized proteins.

For the remaining 334 genes, we found six enriched biological processes (Figure 2D, Table S6). Two processes are related to protein modification (*cellular protein modification process* and *protein phosphorylation*, which represent 8% and 12% of total gene content of these categories, respectively). They concern genes coding for serine/threonine kinases and mitogen-activated kinase (MAPK) (average logFC from 0.97 to 3.08; Table S6). MAPK cascades are involved in filamentation in the budding yeast *Saccharomyces cerevisiae* (production of pseudohyphae), in *Candida albicans* (Liu *et al.* 1993, 1994; Mitchell 1998), and in the phytopathogen *Ustilago maydis* (Mayorga and Gold 1999; Martínez-Espinoza *et al.* 2004). Given these results, we hypothesize that these molecular pathways are also involved in filamentation in *O. novo-ulmi*. The overexpression of these kinases may reflect their role in this cellular differentiation process. Also, the *aminoglycan metabolite process* is enriched in mycelium samples (*P* < 0.01). This category contains 0.2% of the entire genome (17 genes in total and four overexpressed in mycelium). Interestingly, two chitin synthases (OphioH327g0970 and OphioH327g4365.1, logFC 1.08 and 1.34, respectively), one endochitinase (type 1, OphioH327g7070, logFC 1.7), and one anhydro-N-acetylmuramic acid kinase (OphioH327g4206, logFC 3.67) are present in this category (Table S6). Chitin synthases are well known in several fungi to be involved in filamentation, hyphal growth, and conidiation (Borgia *et al.* 1996; Motoyama *et al.* 1996; Martín-Udíroz *et al.* 2004). These roles may be conserved in *O. novo-ulmi*.

In the set of overexpressed genes in the mycelium growth phase, we found nine transcription factors, but none was part of an enriched GO term (Table S6).

#### Carbohydrate-active enzymes and pathogenicity genes:

Using the annotation of CAZymes (carbohydrate-active enzymes) that were described by Cantarel *et al.* (2009) and assigned to *O. novo-ulmi* genes by Comeau *et al.* (2015), we identified 11 genes encoding CAZymes that were overexpressed in the yeast phase and 16 in the mycelial phase (Table 1). Again, for each phase, the set of enzymes that is recruited is different, as yeast cells are enriched in glycoside hydroxylases (GH; *n* = 8) and mycelial growth in glycosyltransferases (GT; *n* = 10). GH are enzymes involved in the breakdown of saccharides, whereas GT are responsible for the biosynthesis of glycosides. Looking at the differentially expressed genes between yeast and mycelial phases, there is very little overlap between the types of CAZymes that are recruited, depending on the growth phase. Thus, yeast and mycelium seem to exert contrasting actions on the carbohydrate component, consistent with the fact that the cell wall of each growth phase is composed of different molecules, a phenomenon that has previously been described in *Candida albicans*, *Histoplasma capsulatum*, and *P. brasiliensis* (Domer *et al.* 1967; Kanetsuna *et al.* 1969; Kanetsuna and Carbonell 1971; Chattaway *et al.* 1973).

**Table 1:** Genes associated with carbohydrate-active enzyme (CAZy) activity in the set of overexpressed genes in yeast or mycelial phase in *Ophiostoma novo-ulmi*

Growth Phase	*O. novo-ulmi* Genes	Overexpression (logFC*^a^* Y *vs.* LM)	Overexpression (logFC Y *vs.* SM)	Description	CAZy*^b^*
Yeast	OphioH327g3368	3.15	2.65	Mannan endo-1,6-alpha-mannosidase DCW1	GH76
	OphioH327g0928	2.65	2.94	Alpha-L-fucosidase	GH29
	OphioH327g0277	1.74	1.16	Glycoside hydrolase 15-related	GH15
	OphioH327g0547	2.41	4.42	1.4-beta-D-glucan cellobiohydrolase C	GH6
	OphioH327g1070	1.38	0.92	Protein phosphatase regulatory subunit	CBM21
	OphioH327g5673	1.28	0.98	Mannan endo-1.6-alpha-mannosidase DCW1 (Defective cell wall 1)	GH76
	OphioH327g4175	1.55	1.95	Endochitinase 2	GH18
	OphioH327g8225	1.55	1.59	Chitin synthase D (GT2 family)	GT2
	OphioH327g2068	1.78	3.19	Xylosidase/arabinosidase (Beta-xylosidase)	GH43
	OphioH327g0647	3.65	3.6	Exoglucanase 1	GH7
	OphioH327g6063	0.94	0.91	UDP-N-acetylglucosaminyltransferase	GT41
Mycelium	OphioH327g0635	2.89	3.18	GDSL-like Lipase/Acylhydrolase	CE12
	OphioH327g3383	1.88	1.20	Carbohydrate-binding module family 52 protein	CBM52
	OphioH327g7070	1.81	1.60	Endochitinase 1 (Complement-fixation antigen)	GH18
	OphioH327g6213	1.42	1.48	Glycosyltransferase family 22 protein	GT22
	OphioH327g7370	4.60	3.68	Endo-N-acetyl-beta-D-glucosaminidase	GH18
	OphioH327g4365.1	1.38	1.30	Chitin synthase 3	GT2
	OphioH327g3285	1.30	1.64	Protein mannosyltransferase 1	GT39
	OphioH327g6864	1.41	1.66	Beta-glucanosyltransferase gel2	GH72
	OphioH327g7251	3.66	3.85	Putative cellulose synthase 3 (Cyclic di-GMP-binding domain)	GT2
	OphioH327g5590	4.86	5.79	Rhamnogalacturonase	GH28
	OphioH327g8280	1.24	1.42	UDP-glucose:glycoprotein glucosyltransferase	GT24
	OphioH327g5097	1.10	1.20	Dolichol-phosphate mannosyltransferase	GT2
	OphioH327g2358	0.90	1.39	Oligosaccharyl transferase subunit	GT66
	OphioH327g3447	1.73	1.99	EGF domain-specific O-linked N-acetylglucosamine transferase	GT61
	OphioH327g4450	0.91	1.03	Mannosyltransferase pmti	GT39
	OphioH327g0970	0.93	1.24	Chitin synthase 1 (Class-III chitin synthase 3)	GT2

a LogFC, log_2_ of fold change between yeast and mycelium phases; Y, yeast phase; LM, mycelium grown in liquid medium; SM, mycelium grown in Petri dishes. FDR ≤1%.

b GH, glycoside hydrolases; GT, glycosyltransferases; CBM, carbohydrate-binding module. Numbers associated with CAZy types refer to the family number.

In their study on *O. novo-ulmi* annotation, Comeau and colleagues identified 1731 genes as homologs of genes that are involved in pathogen/host interactions and as part of the PHI-base, a database that catalogs genes that are related to pathogenicity and virulence for fungal, oomycetes, and bacterial pathogens (www.phi-base.org) (Winnenburg *et al.* 2008; Comeau *et al.* 2015). Here, we found that 217 of these genes are differentially expressed between yeast and mycelial phases (107 overexpressed in the yeast phase and 110 in the mycelial phase; Table S5 and Table S6). Although cultures that were subjected to RNAseq analysis were grown under axenic conditions, these genes represent many opportunities to link each growth phase of *O. novo-ulmi* to pathogenicity.

### Comparison of *Ophiostoma novo-ulmi* transcriptomes with those of other dimorphic pathogens

To systematically assess the molecular similarities between *O. novo-ulmi* and well-established model species for the study of dimorphism, we compared our transcriptomic data with previously published data for *Candida albicans* (Bruno *et al.* 2010) and *Histoplasma capsulatum* (Edwards *et al.* 2013), which are both ascomycete dimorphic human pathogens (Figure 1). Given that these three fungi are dimorphic, we expect many overlaps regarding regulation of phase-dependent gene expression.

**Figure 3:**
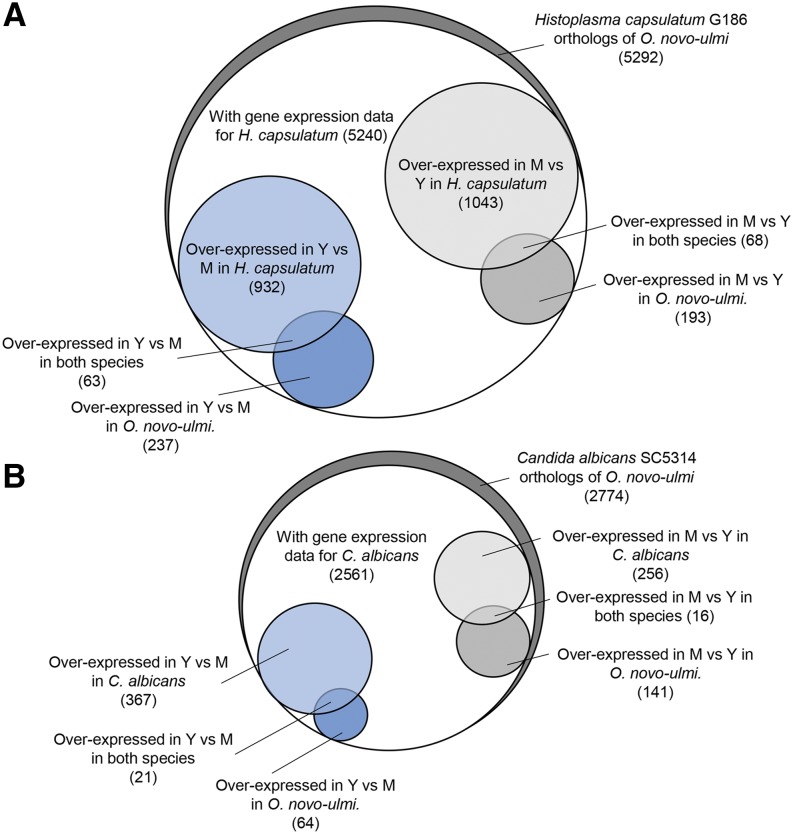
Comparison of gene expression using orthologous genes found between *Ophiostoma novo-ulmi* and (A) *Histoplasma capsulatum* or (B) *Candida albicans*. Y, yeast; M, mycelium. Number of genes are indicated between parentheses.

In their study, Edwards *et al.* (2013) sequenced mRNA extracted from distinct yeast and mycelial growth phases of *H. capsulatum*. Yeast cells were grown to late exponential phase at 37° with agitation, whereas the mycelium was produced at 25°. As the values and gene names that are presented in this 2013 study corresponded to a *de novo* assembly from transcriptomes mapped onto the reference genome, we compared the complete set of O. *novo-ulmi* exons with the *de novo* assembly of G186 using *tblastx*. We found 5292 unique *de novo*–assembled *H. capsulatum* genes that matched *O. novo-ulmi* genes (Table S3, Figure 3A). The percentage of nucleotide sequence identity for the orthologous pairs ranged from 19.75 to 100% (average identity, 60.87%) (Figure S3).

We used the transcriptome dataset of *H. capsulatum* to compare gene expression levels between *H. capsulatum* and *O. novo-ulmi*. By reanalyzing RNAseq data from Edwards *et al.* (2013) with our workflow, we determined that 5240 orthologs of *O. novo-ulmi* are expressed in *H. capsulatum*. However, less than 1.5% of orthologous genes are overexpressed in yeast or in mycelium in both species (Figure 3A, Table S7, Table S8). Thus, the overlap represents only a few orthologs and no more than 6.5% of the differentially expressed genes between yeast and mycelium phases of *O. novo-ulmi*. The overlap is greater than expected by chance in the mycelium phase only (*P* = 0.015) (Table 2), suggesting that the expression profiles are conserved. Nevertheless, we still found some evidence of specific gene conservation in the yeast growth phase. Indeed, the gene encoding the aforementioned urease (OphioH327g2055) is orthologous to a *H. capsulatum* gene (HC186_00717.T1), which is also overexpressed in the yeast phase (Table S8). This supports our hypothesis of a conserved role for urease across fungal pathogens. In terms of biological processes, the small overlapping gene sets are not very representative of the diversity found in *Ophiostoma novo-ulmi* (Table 3). Only three biological processes for yeast were enriched (none for the mycelium). All of these processes are involved in the transport of ions (four genes) and cations (eight genes). It has been shown that genes involved in sulfur metabolism are overexpressed in the yeast phase of *H. capsulatum* (Hwang *et al.* 2003). Because this process is not enriched in *O. novo-ulmi*, it seems that this metabolic pathway is not as important in *O. novo-ulmi* as it is in *H. capsulatum*. Overall, these results highlight the occurrence of different molecular profiles for yeast and mycelial growth phases, depending on the species. *Histoplasma capsulatum* is a thermally sensitive fungus that switches from a virulent mycelium to pathogenic yeasts at 37° (Maresca and Kobayashi 1989). Consequently, the growth conditions of yeast phases are distinct between *O. novo-ulmi* and *H. capsulatum*. The optimal growth temperature of *O. novo-ulmi* is 20–22° (Brasier 1991), and it grows well in both phases from 20° to 32° (data not shown). Thus, the ecological niches that are filled by these two fungi are distinct; both phylogenetic distance and the different ecological niches could explain most of the differences highlighted here.

**Table 2:** Significance of the overlap of overexpressed orthologs in yeast and mycelium with respect to the species comparison for *Ophiostoma novo-ulmi*, *Histoplasma capsulatum*, and *Candida albicans*

Growth Phase	Species Compared	Overlap	*P*-value*^a^*
Yeast	*H. capsulatum* and *O. novo-ulmi*	63	0.1406
	*C. albicans* and *O. novo-ulmi*	21	0.0023
	*H. capsulatum* and *C. albicans*	53	0.7543
Mycelium	*H. capsulatum* and *O. novo-ulmi*	68	0.0149
	*C. albicans* and *O. novo-ulmi*	16	0.3688
	*H. capsulatum* and *C. albicans*	54	0.0182

a *P-values* calculated with the hypergeometric test indicate the probability of having an overlap greater than the observed value.

**Table 3:** Enriched GO terms (biological processes) in yeast for orthologs that are overexpressed in both *Ophiostoma novo-ulmi* and *Histoplasma capsulatum*

Growth Phase	GO Accession	Term Representation in Gene Set (%)*^a^*	Term representation in genome (%)*^b^*	*P*-value*^c^*	Term
Yeast	GO:0015672	9.52	0.49	0.0006	Monovalent inorganic cation transport
	GO:0030001	7.84	0.59	0.0016	Metal ion transport
	GO:0006811	4.54	2.04	0.0003	Ion transport

a Term representation in gene set: percentage of genes associated with a given term that are found in the set of yeast overexpressed genes.

b Term representation in genome: percentage of the whole genome that is associated with a given term.

c *P-values* obtained using GOseq package.

We also compared *O. novo-ulmi* yeast–mycelium differential expression data with those obtained for the same phases in *Candida albicans*. As part of a large transcriptome analysis in *C. albicans*, two biological replicates for each of the yeast and mycelium growth phases were obtained using media incorporating serum at 37° (mycelium) or not incorporating serum at 30° (yeasts) (Bruno *et al.* 2010). We found 2774 *C. albicans* genes that had an ortholog in *O. novo-ulmi* genes (Figure 3B, Table S3). Here the range of nucleotide sequence identity percentages for each orthologous pair is between 16.54% and 100% (average identity, 53.27%) (Figure S3). Because *C. albicans* is from the class Saccharomycetes, we expected a number of orthologs that was close to the one associated with *Saccharomyces cerevisiae* (3179) (Table S3). We determined that 2561 orthologs of *Ophiostoma* are expressed in *C. albicans* and only 21 genes are overexpressed in yeast and 16 genes are overexpressed in mycelium in both species (respectively, 0.8% and 0.6% of the total number of expressed orthologs) (Table S9). Here, only yeast growth phase overlap is significant (*P* = 0.0023) (Table 2). The few orthologous genes that show the same regulation pattern between the two species may be conserved, but the small number may be related to the phylogenetic distance and the different growth conditions that allow yeast or mycelium to develop. In fact, increased temperature coupled with other conditions (presence of serum, pH modification) triggers filamentation in *C. albicans* (Odds 1988).

Interestingly, of more than the 3391 orthologs that were shared between *H. capsulatum* and *C. albicans* (average sequence identity, 53.94%) (Table S3, Figure S3), only 53 are overexpressed in the yeast phase of both species and 54 are overexpressed in mycelium (data not shown). Again, the overlap is statistically significant (*P* = 0.018) for only one growth phase, *i.e.*, the mycelium (Table 2). Thus, it would appear that even thermally regulated species may have established different strategies to grow either in yeast form or in mycelium phase. As previously mentioned, *H. capsulatum* and *C. albicans* have contrasting responses to temperature as incubation at 37° induces yeast growth in the former but triggers filamentation in the latter. Moreover, in *C. albicans*, both phases are found at 37°; it is the combination of multiple factors that determine which growth phase will be favored (Odds 1988; Mitchell 1998). In the study by Bruno *et al.* (2010), filamentation was induced by adding serum to the medium at 37°. Thus, sharing the same host is not sufficient to induce similar regulation of the expression of orthologs. In this case, the phylogenetic distance between *H. capsulatum* and *C. albicans* may also account for the small degree of overlap between overexpressed orthologs in yeast and mycelial phases. With the development of current technologies, closer plant and human pathogen models may be developed in the future and allow us to better understand how filamentation evolved and its relationship with ecology, for instance, in the phylogenetic group of *O. novo-ulmi*. For example, *Sporothrix schenckii* would be an excellent candidate for comparison with *O. novo-ulmi* because they share 7171 orthologs (Figure 1, Table S3), but they infect different hosts and show distinct ecological niches.

### Conclusions

Our study provides insights into the molecular functions and gene expression profiles that are involved in fungal growth processes. It also highlights strategies that are related to yeast or mycelium phases in *O. novo-ulmi*, a nonthermally regulated dimorphic fungus. We showed that these phases display different transcriptome signatures and that some biological processes, such as protein modification or amino acid metabolism, are specific to one of the two phases. Further, carbohydrate-active enzymes seem to be differentially involved in distinct growth phases. Some of these functions are already described for a limited number of fungal species and seem to be conserved in *O. novo-ulmi*, including MAP kinases, catalases, chitinases, and ureases. By comparing transcriptomes of *C. albicans* and *H. capsulatum* with our data, we found very little overlap and no conserved biological processes. Overall, we show that *O. novo-ulmi* possesses specific gene regulation processes and that comparisons with well-studied species do not cover most of the transcriptomic variation that was observed between the yeast and mycelium growth phases. Given that most of the model species used for studying dimorphism are human pathogens, expanding our knowledge of an ascomycete plant pathogen is greatly needed. *O. novo-ulmi* is a perfect candidate with the ability to manipulate dimorphism *in vitro* (Naruzawa and Bernier 2014) and a genome that has already been sequenced and, now, for which large-scale transcriptomic data are available.

## 

## Supplementary Material

Supporting Information
