# Supplementary material for: RNAseq Analysis Highlights Specific Transcriptome Signatures of Yeast and Mycelial Growth Phases in the Dutch Elm Disease Fungus Ophiostoma novo-ulmi
Source: G3 (Bethesda). 2015 Sep 17;5(11):2487–95. doi: 10.1534/g3.115.021022 (PMC4632067; doi:10.1534/g3.115.021022)
Supplement: Supporting Information [file supp_5_11_2487__index.html]

RNAseq Analysis Highlights Specific Transcriptome Signatures of Yeast and Mycelial Growth Phases in the Dutch Elm Disease Fungus Ophiostoma novo-ulmi — Supporting Information 

# RNAseq Analysis Highlights Specific Transcriptome Signatures of Yeast and Mycelial Growth Phases in the Dutch Elm Disease Fungus *Ophiostoma novo-ulmi*

## Supporting Information for Nigg *et al.*, 2015

**Files in this Data Supplement:**

- Supporting Information - Figures S1-S3 and Tables S1-S9 (PDF, 1 MB)
- Figure S1 - Workflow for RNAseq library preparation, cleaning and analysis for the 3 growth conditions (yeasts, mycelium on petri dishes and mycelium in flask, 3 replicates per condition) for *Ophiostoma novo-ulmi*. (PDF, 485 KB)
- Figure S2 - Number of *Ophiostoma novo-ulmi* RNAseq reads per sample and per gene. (PDF, 324 KB)
- Figure S3 - Distribution of the number of orthologs between two species per percentage of gene sequence identity. (PDF, 391 KB)
- Table S1 - Species and strain/isolate names, references and sequence sizes for the species used to build the phylogenetic tree. (PDF, 118 KB)
- Table S2 - General characteristics for each RNAseq sample for *Ophiostoma novo-ulmi*. (PDF, 156 KB)
- Table S3 - Number of orthologous genes found with the reciprocal best blast hits (RBH) method compared with the Inparanoid method of Khoshraftar et al. (2013). (PDF, 116 KB)
- Table S9 - Description of the 21 orthologous genes overexpressed in yeast in both *Ophiostoma novo-ulmi* and *Candida albicans*. (PDF, 151 KB)
- Table S4 - Read counts for each gene per sample in *Ophiostoma novo-ulmi*. (.xlsx, 598 KB)
- Table S5 - Genes overexpressed in yeast phase of *Ophiostoma novo-ulmi*. (.xlsx, 46 KB)
- Table S6 - Genes overexpressed in mycelium phase of *Ophiostoma novo-ulmi*. (.xlsx, 43 KB)
- Table S7 - Description of the 63 orthologous genes overexpressed in yeast in both *Ophiostoma novo-ulmi* and *Histoplasma capsulatum*. (.xlsx, 12 KB)
- Table S8 - Description of the 68 orthologous genes overexpressed in mycelium in both *Ophiostoma novo-ulmi* and *Histoplasma capsulatum*. (.xlsx, 13 KB)
