# Supplementary material for: RNAseq Analysis Highlights Specific Transcriptome Signatures of Yeast and Mycelial Growth Phases in the Dutch Elm Disease Fungus Ophiostoma novo-ulmi
Source: G3 (Bethesda). 2015 Sep 17;5(11):2487–95. doi: 10.1534/g3.115.021022 (PMC4632067; doi:10.1534/g3.115.021022)
Supplement: Supporting Information [file supp_g3.115.021022_021022SI.pdf]

G3·Genes | Genomes | Genetics

RNAseq analysis highlights specific transcriptome signatures of yeast and mycelial growth phases in the Dutch elm disease fungus *Ophiostoma novo-ulmi*.

Martha Nigg [*, ƚ], Jérôme Laroche [*, ǂ], Christian R. Landry [*, §] and Louis Bernier [*, ƚ]

[*] Institut de Biologie Intégrative et des Systèmes (IBIS), Université Laval, Québec, G1V 0A6, Canada

[ƚ] Centre d'Étude de la Forêt (CEF) and Département des sciences du bois et de la forêt, Université Laval, Québec, G1V 0A6, Canada

[ǂ] Plateforme de bio-informatique, Institut de Biologie Intégrative et des Systèmes (IBIS), Université Laval, Québec, G1V 0A6, Canada

[§] Département de biologie, Université Laval, Québec, G1V 0A6, Canada

Data availability: all RNAseq sequences are available under the NCBI BioProject (PRJNA260920) on Genbank.

Corresponding author: Martha Nigg,
Centre d'Étude de la Forêt (CEF)
Département des sciences du bois et de la forêt
Institut de Biologie Intégrative et des Systèmes (IBIS)
Room 2255, Pavillon Charles-Eugène-Marchand
1030, Avenue de la Médecine
Université Laval
Québec (Québec) G1V 0A6
Canada

Email: martha.nigg.1@ulaval.ca

DOI: 10.1534/g3.115.021022

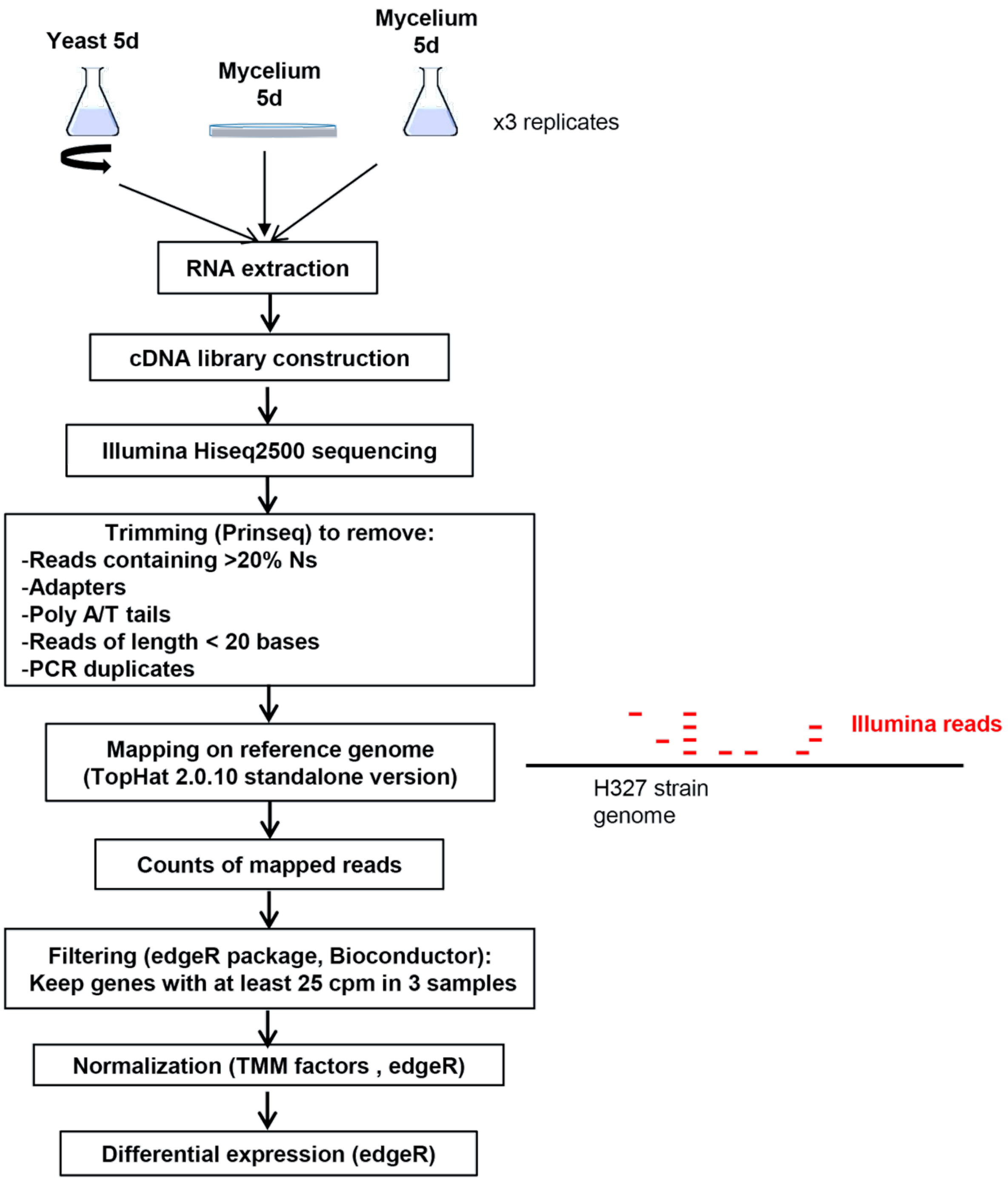

Figure S1 Workflow for RNAseq library preparation, cleaning and analysis for the three growth conditions (yeasts, mycelium on petri dishes and mycelium in flask, three replicates per condition) for *Ophiostoma novo-ulmi*.

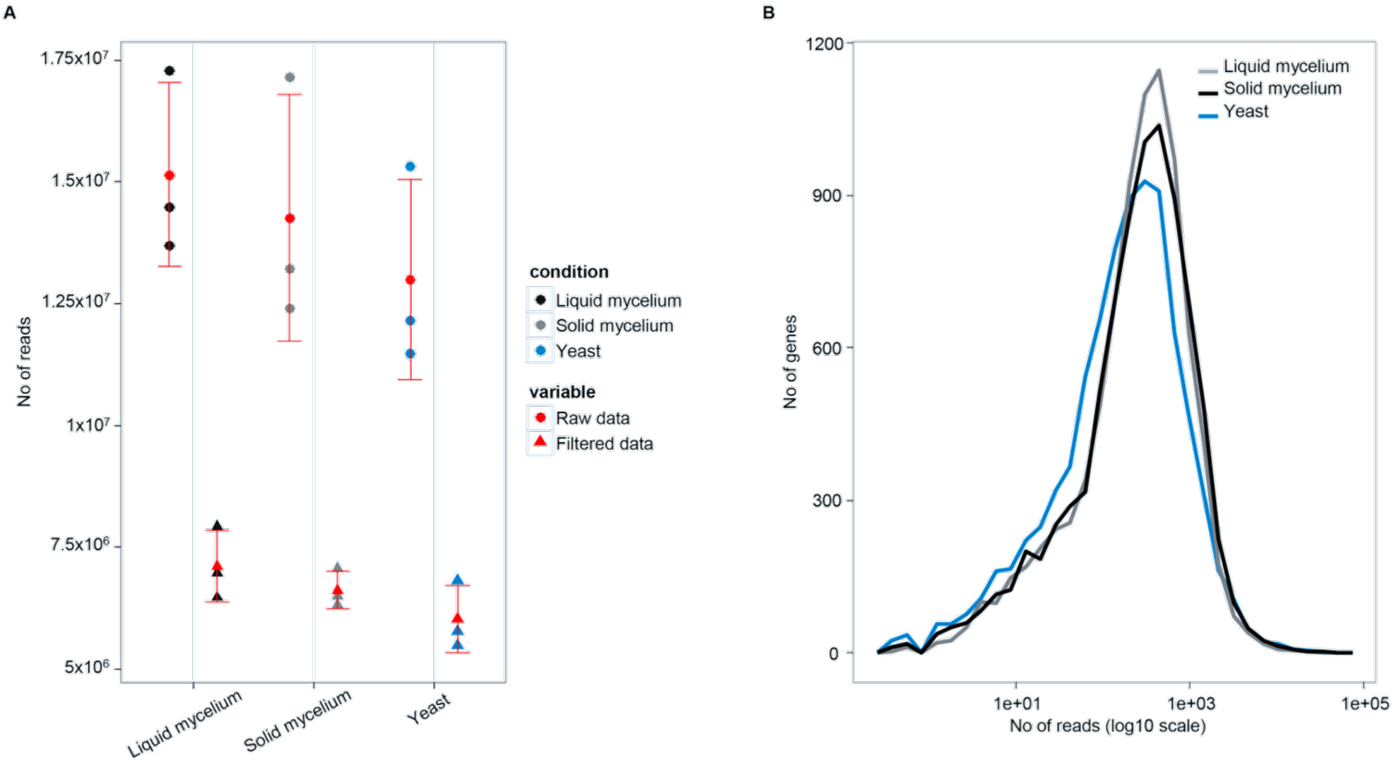

Figure S2 Number of *Ophiostoma novo-ulmi* RNAseq reads per sample and per gene: (A) Number of reads before (raw reads, dot) and after filtration/cleaning (filtered reads without duplicates, triangle) process present in each of the three conditions. Red dots: means of the three repetitions with standard deviation. No significant differences between conditions for each variable (Fisher's exact test). (B) Distribution of the number of reads (log_{10} scale) per genes, per conditions (mean of three replicates).

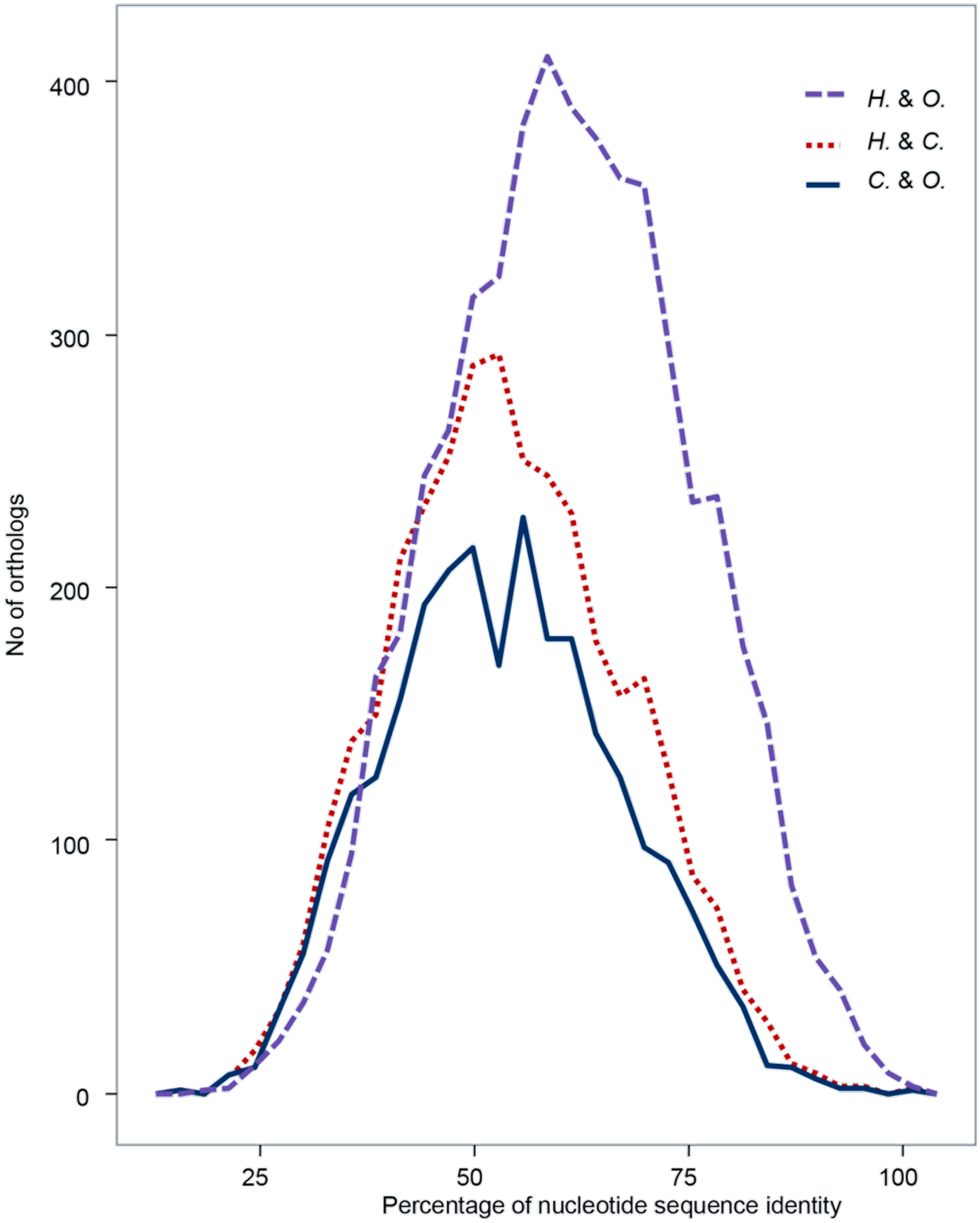

Figure S3 Distribution of the number of orthologs between two species per percentage of gene sequence identity. *H.*: *Histoplasma capsulatum*; *C.*: *Candida albicans*; *O.*: *Ophiostoma novo-ulmi*

Table S1 Species and strain/isolate names, references and sequence sizes for the species used to build the phylogenetic tree.

Species name	Strain/isolate	Reference	Sequence size (nt)
Neurospora crassa	WA0000019131	Palowska *et al.*, 2014 The diversity of endophytic fungi in the above-ground tissue of two *Lycopodium* species in Poland. Symbiosis 63:(2)87-97	198
Aspergillus nidulans	YIMPH30005	Miao *et al.*, 2015 Rhizospheric fungi of *Panax notoginseng*: diversity and antagonism to host phytopathogens. Journal of Ginseng Research, http://dx.doi.org/10.1016/j.jgr.2015.06.004	211
Candida albicans	SC5314	Diezmann and Dietrich (unpublished) Natural Variation in *Candida* and *Saccharomyces*	204
Saccharomyces cerevisiae	S288c	Diezmann and Dietrich (unpublished) Natural Variation in *Candida* and *Saccharomyces*	204
Paracoccidioides brasiliensis	-	Kasuga, White and Taylor (unpublished) *Ajellomyces* ITS	209
Sporothrix schenckii	C9862	Camacho *et al*., 2015 Molecular epidemiology of human sporotrichosis in Venezuela reveals high frequency of *Sporothrix globosa*. BMC Infect. Dis. 15:(1)839	209
Ophiostoma novo-ulmi	H327	Naruzawa and Bernier, 2014 Control of yeast-mycelium dimorphism in vitro in Dutch elm disease fungi by manipulation of specific external stimuli. Fungal Biol 118:(11)872-884	214
Ophiostoma ulmi	W9	Naruzawa and Bernier, 2014 Control of yeast-mycelium dimorphism in vitro in Dutch elm disease fungi by manipulation of specific external stimuli. Fungal Biol 118:(11)872-884	215
Histoplasma capsulatus	VPCI 881/P/13	Kathuria *et al*., (unpublished) *Histoplasma capsulatum* and histoplasmosis: A review	210
Taphrina deformans	NRRL T-470	Rodrigues and Fonseca, 2003 Molecular systematics of the dimorphic ascomycete genus *Taphrina*. Int. J. Syst. Evol. Microbiol. 53:(PT 2)607-616	216

Table S2 General characteristics for each RNAseq sample for *Ophiostoma novo-ulmi*.

Conditions	Yeast						Solid mycelium						Liquid mycelium					
Replicates	1		2		3		1		2		3		1		2		3	
Duplicates trimmed	No	Yes	No	Yes	No	Yes	No	Yes	No	Yes	No	Yes	No	Yes	No	Yes	No	Yes
#[a] Reads after trimming	1.52E+07	6.81E+06	1.14E+07	5.49E+06	1.21E+07	5.77E+06	1.24E+07	6.31E+06	1.30E+07	6.48E+06	1.71E+07	7.05E+06	1.72E+07	7.92E+06	1.44E+07	6.97E+06	1.36E+07	6.46E+06
# Mapped reads	1.01E+07	4.53E+06	7.59E+06	3.69E+06	7.67E+06	3.79E+06	7.37E+06	4.13E+06	7.92E+06	4.26E+06	9.46E+06	4.22E+06	1.08E+07	5.23E+06	8.92E+06	4.55E+06	8.70E+06	4.28E+06
# Mapped bases	9.87E+08	4.30E+08	7.42E+08	3.51E+08	7.51E+08	3.62E+08	7.21E+08	3.96E+08	7.75E+08	4.07E+08	9.25E+08	3.99E+08	1.06E+09	4.98E+08	8.72E+08	4.33E+08	8.51E+08	4.08E+08
Mean read length	97.87	94.98	97.81	95.21	97.81	95.32	97.82	95.80	97.81	95.63	97.77	94.52	97.82	95.20	97.75	95.30	97.83	95.31
Exons coverage depth	66.26	28.91	49.84	23.60	50.41	24.29	48.43	26.56	52.04	27.34	62.12	26.78	70.93	33.44	58.55	29.10	57.16	27.42
Genome coverage depth	30.04	13.10	22.59	10.70	22.85	11.01	21.96	12.04	23.59	12.40	28.16	12.14	32.16	15.16	26.54	13.19	25.91	12.43
EdgeR normalization factor	0.68	0.84	0.71	0.82	0.85	0.94	1.25	1.15	1.13	1.09	1.28	1.12	1.03	1.04	1.07	1.07	0.90	0.97
# Genes with at least 1 read	8175	8296	8144	8257	8143	8241	8194	8316	8199	8318	8200	8322	8200	8325	8189	8304	8175	8287

[a] #=number

Table S3 Number of orthologous genes found with the reciprocal best blast hits (RBH) method compared with the Inparanoid method of Khoshraftar *et al*. (2013). The number of genes in each species is indicated between parentheses.

Species compared (no of genes)	RBH method	Khoshraftar *et al.* 2013
Ophiostoma ulmi (8639)/ *Neurospora crassa* (9730)	6276	5517
O. ulmi/ Saccharomyces cerevisiae (6604)	3179	2483
O. ulmi/ O. novo-ulmi (8640)	8220	NA[a]
O. novo-ulmi/ N. crassa	6360	NA
O. novo-ulmi/ S. cerevisiae	3260	NA
O. novo-ulmi/ Sporothrix schenckii (8674)	7171	NA
O. novo-ulmi/ Candida albicans (6218)	2774	NA
O. novo-ulmi/ Histoplasma capsulatum (9233)	5292	NA
H. capsulatum / *C. albicans*	3391	NA

[a] NA : data Not Available

Tables S4-S8

Available for download as excel tables at www.g3journal.org/lookup/suppl/doi:10.1534/g3.115.021022/-/DC1

Table S4: Read counts for each gene per sample in *Ophiostoma novo-ulmi*

Table S5: Genes overexpressed in yeast phase of *Ophiostoma novo-ulmi*

Table S6: Genes overexpressed in mycelium phase of *Ophiostoma novo-ulmi*

Table S7: Description of the 63 orthologous genes overexpressed in yeast in both *Ophiostoma novo-ulmi* and *Histoplasma capsulatum*

Table S8: Description of the 68 orthologous genes overexpressed in mycelium in both *Ophiostoma novo-ulmi* and *Histoplasma capsulatum*

Table S9 Description of the 21 orthologous genes overexpressed in yeast in both *Ophiostoma novo-ulmi* and *Candida albicans*. LogFC: average log of fold change between yeast and mycelium phases. FDR ≤ 0.01.

Growth phase	*O. novo-ulmi* genes	Over-expression (logFC)	Description	Candida orthologs	Over-expression (logFC *in C. albicans*)
Yeast	OphioH327g3645	2.76	Glutamate_dehydrogenase	C4_06120W_B	1.38
	OphioH327g4970	3.7	NOL1_NOP2_SUN_domain_containing_protein	CR_02030C_A	0.63
	OphioH327g3717	3.665	Aquaporin-1	CR_02920C_B	1.48
	OphioH327g2293	3.225	Denitrification_regulatory_protein_nirq	C4_00970C_B	0.68
	OphioH327g4808	2.845	Cytosine_deaminase	C6_00620W_B	0.78
	OphioH327g0568	1.93	ATP-dependent_RNA_helicase_ded1	C3_06100C_A	0.71
	OphioH327g2187	1.985	Flocculation_suppression_protein_(Protein_SFL1)	CR_05990C_B	1.15
	OphioH327g2220	2.175	RNA-binding_protein	C5_00790C_A	0.80
	OphioH327g8083	1.76	Low-temperature_viability_protein_ltv1	CR_10650W_A	0.71
	OphioH327g5596	1.71	U3_small_nucleolar_RNA-associated_protein_sof1	C3_00560C_A	0.60
	OphioH327g5986	2.04	Serine/threonine-protein_kinase_srk1_(Sty1-regulated_kinase_1)	C2_07130C_A	0.57
	OphioH327g5054	2.34	Hsp70/Hsp90_co-chaperone_CNS1_(Cyclophilin_seven_suppressor_1/STI1_stress-inducible_protein_homolog)	C1_00560W_A	0.66
	OphioH327g3374	2.28	Fluconazole_resistance_protein_1	C3_06850W_A	1.89
	OphioH327g5556	1.565	DNA-directed_RNA_polymerase,_mitochondrial	C1_00640C_B	0.64
	OphioH327g4855	2.365	U3_small_nucleolar_RNA-associated_protein_20_(U3_snoRNA-associated_protein_20)	C3_01200W_B	0.89
	OphioH327g7825	1.43	RNA_recognition_domain-containing_protein	C3_05150W_B	0.73
	OphioH327g0412	1.255	Fimbrin_(ABP67)	C6_02730W_A	0.50
	OphioH327g7377	2.235	Major_facilitator_superfamily_transporter_multidrug_resistance	C6_04610C_A	0.87
	OphioH327g8261	1.65	Integral_membrane_protein	C1_10360C_A	1.17
	OphioH327g1355	1.38	U3_small_nucleolar_ribonucleoprotein_protein_mpp10	C2_00070C_B	0.60
	OphioH327g5712	1.22	P-type_ATPase	C2_02490C_B	0.76
Mycelium	OphioH327g6748	3.285	N-acetyltransferase-like_protein	C6_00140C_A	0.94
	OphioH327g3302	2.48	IMP-specific_5'-nucleotidase_1	C1_01650W_B	0.72
	OphioH327g0724	2.25	Putative_uncharacterized_protein	CR_09930W_B	0.71
	OphioH327g3285	2.12	Protein_mannosyltransferase_1	C7_02890C_B	0.67
	OphioH327g8280	1.95	UDP-glucose:glycoprotein_glucosyltransferase	C3_02960C_B	0.85
	OphioH327g8395	1.785	Mitogen-activated_protein_kinase	C4_06480C_B	2.05
	OphioH327g4472	1.76	Aromatic_aminotransferase	C2_00340C_A	0.75

OphioH327g5097	1.7	Dolichol-phosphate_mannosyltransferase	C1_08010W_B	0.75
OphioH327g8526	1.655	Rho-GDP_dissociation_inhibitor	C3_05000W_A	1.00
OphioH327g4520	1.75	Ornithine_carbamoyltransferase-like_protein	C6_03230W_B	1.17
OphioH327g6717	1.58	Succinyl-3-ketoacid-coenzyme_a_transferase	C2_07240C_B	1.21
OphioH327g6465	1.61	Fungal_specific_transcription_factor	CR_09210W_A	1.50
OphioH327g2358	1.595	Oligosaccharyl_transferase_subunit	C2_01670C_B	0.74
OphioH327g0333	1.3	Crotonase	C1_03320C_B	0.71
OphioH327g2998	2.545	Aspartic-type_endopeptidase	CR_07800W_B	1.48
OphioH327g4450	1.425	Mannosyltransferase_pmti	C2_06100W_B	0.98
