# Supplementary material for: RNAseq Analysis Highlights Specific Transcriptome Signatures of Yeast and Mycelial Growth Phases in the Dutch Elm Disease Fungus Ophiostoma novo-ulmi
Source: G3 (Bethesda). 2015 Sep 17;5(11):2487–95. doi: 10.1534/g3.115.021022 (PMC4632067; doi:10.1534/g3.115.021022)
Supplement: Supporting Information [file supp_g3.115.021022_FigureS1.pdf]

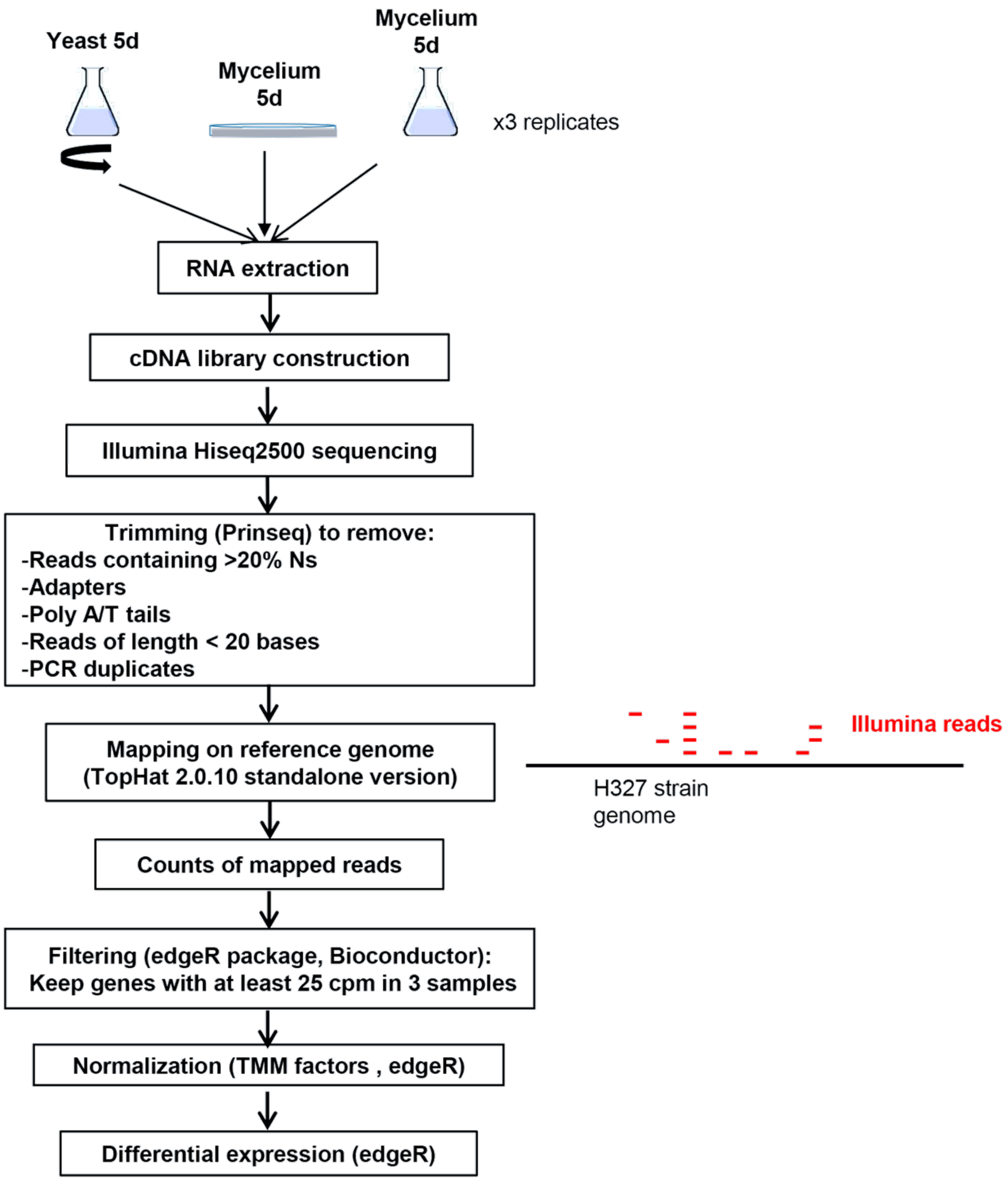

Figure S1 Workflow for RNAseq library preparation, cleaning and analysis for the three growth conditions (yeasts, mycelium on petri dishes and mycelium in flask, three replicates per condition) for *Ophiostoma novo-ulmi*.
