# Supplementary material for: RNAseq Analysis Highlights Specific Transcriptome Signatures of Yeast and Mycelial Growth Phases in the Dutch Elm Disease Fungus Ophiostoma novo-ulmi
Source: G3 (Bethesda). 2015 Sep 17;5(11):2487–95. doi: 10.1534/g3.115.021022 (PMC4632067; doi:10.1534/g3.115.021022)
Supplement: Supporting Information [file supp_g3.115.021022_FigureS2.pdf]

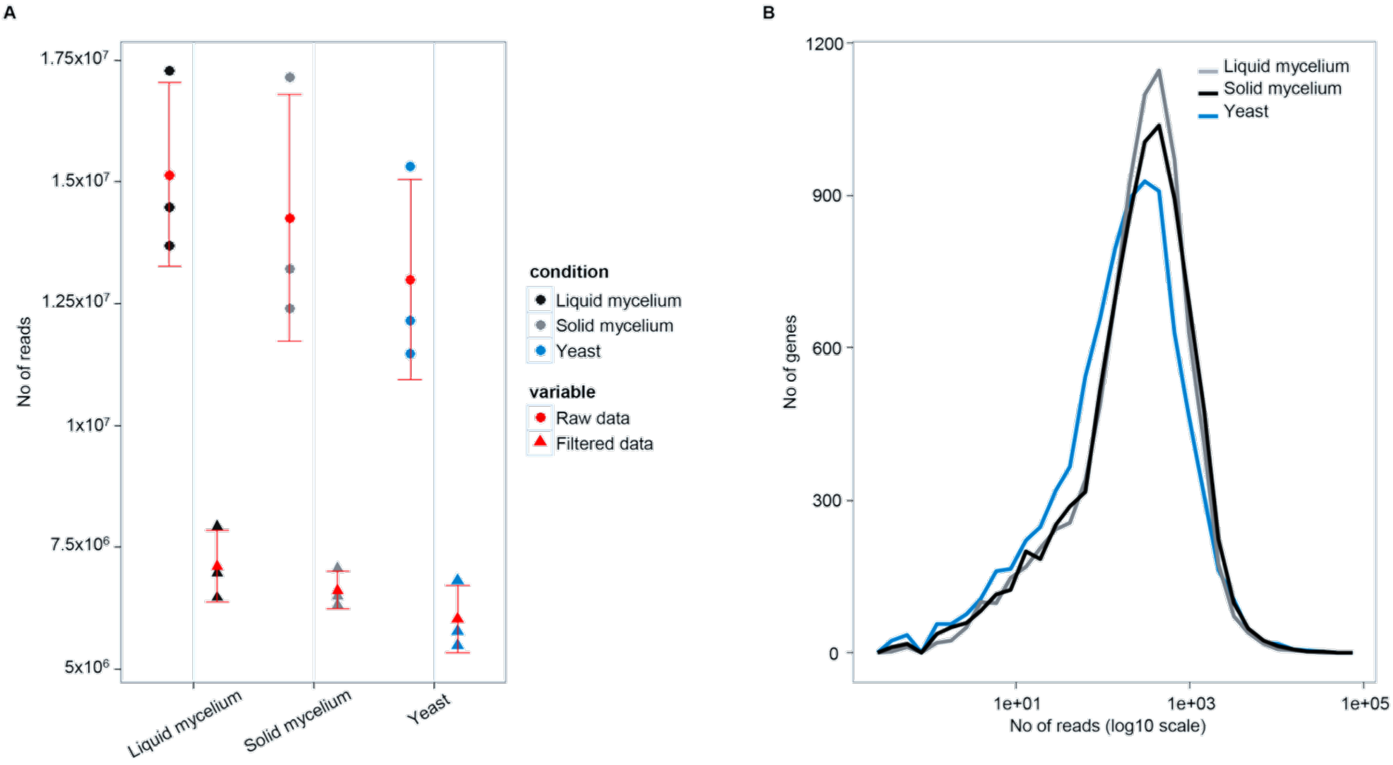

Figure S2 Number of *Ophiostoma novo-ulmi* RNAseq reads per sample and per gene: (A) Number of reads before (raw reads, dot) and after filtration/cleaning (filtered reads without duplicates, triangle) process present in each of the three conditions. Red dots: means of the three repetitions with standard deviation. No significant differences between conditions for each variable (Fisher's exact test). (B) Distribution of the number of reads (log_{10} scale) per genes, per conditions (mean of three replicates).
