# Supplementary material for: RNAseq Analysis Highlights Specific Transcriptome Signatures of Yeast and Mycelial Growth Phases in the Dutch Elm Disease Fungus Ophiostoma novo-ulmi
Source: G3 (Bethesda). 2015 Sep 17;5(11):2487–95. doi: 10.1534/g3.115.021022 (PMC4632067; doi:10.1534/g3.115.021022)
Supplement: Supporting Information [file supp_g3.115.021022_FigureS3.pdf]

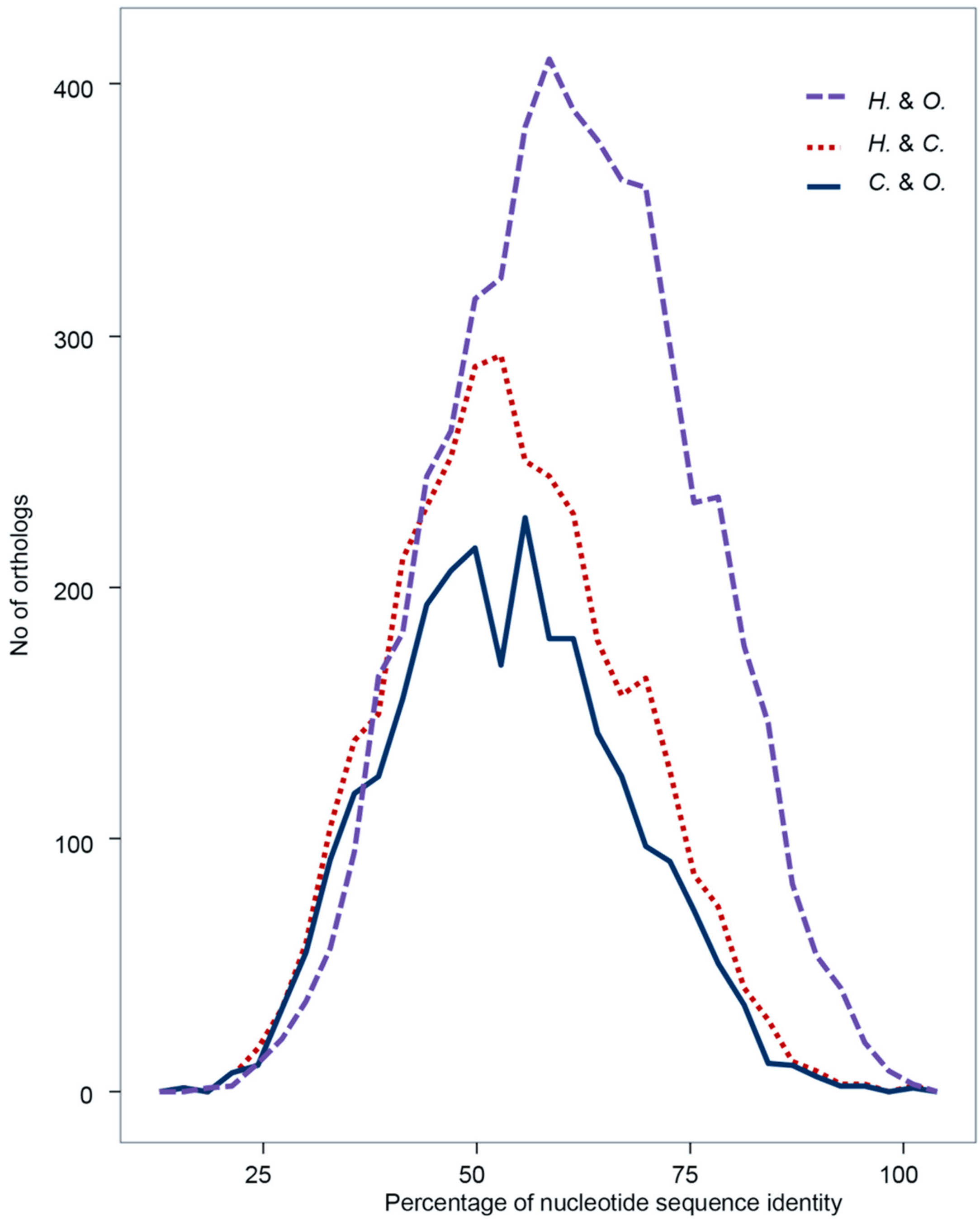

Figure S3 Distribution of the number of orthologs between two species per percentage of gene sequence identity. *H.*: *Histoplasma capsulatum*; *C.*: *Candida albicans*; *O.*: *Ophiostoma novo-ulmi*
