# Supplementary material for: RNAseq Analysis Highlights Specific Transcriptome Signatures of Yeast and Mycelial Growth Phases in the Dutch Elm Disease Fungus Ophiostoma novo-ulmi
Source: G3 (Bethesda). 2015 Sep 17;5(11):2487–95. doi: 10.1534/g3.115.021022 (PMC4632067; doi:10.1534/g3.115.021022)
Supplement: Supporting Information [file supp_g3.115.021022_TableS1.pdf]

Table S1 Species and strain/isolate names, references and sequence sizes for the species used to build the phylogenetic tree.

Species name	Strain/isolate	Reference	Sequence size (nt)
Neurospora crassa	WA0000019131	Palowska *et al.*, 2014 The diversity of endophytic fungi in the above-ground tissue of two *Lycopodium* species in Poland. Symbiosis 63:(2)87-97	198
Aspergillus nidulans	YIMPH30005	Miao *et al.*, 2015 Rhizospheric fungi of *Panax notoginseng*: diversity and antagonism to host phytopathogens. Journal of Ginseng Research, http://dx.doi.org/10.1016/j.jgr.2015.06.004	211
Candida albicans	SC5314	Diezmann and Dietrich (unpublished) Natural Variation in *Candida* and *Saccharomyces*	204
Saccharomyces cerevisiae	S288c	Diezmann and Dietrich (unpublished) Natural Variation in *Candida* and *Saccharomyces*	204
Paracoccidioides brasiliensis	-	Kasuga, White and Taylor (unpublished) *Ajellomyces* ITS	209
Sporothrix schenckii	C9862	Camacho *et al*., 2015 Molecular epidemiology of human sporotrichosis in Venezuela reveals high frequency of *Sporothrix globosa*. BMC Infect. Dis. 15:(1)839	209
Ophiostoma novo-ulmi	H327	Naruzawa and Bernier, 2014 Control of yeast-mycelium dimorphism in vitro in Dutch elm disease fungi by manipulation of specific external stimuli. Fungal Biol 118:(11)872-884	214
Ophiostoma ulmi	W9	Naruzawa and Bernier, 2014 Control of yeast-mycelium dimorphism in vitro in Dutch elm disease fungi by manipulation of specific external stimuli. Fungal Biol 118:(11)872-884	215
Histoplasma capsulatus	VPCI 881/P/13	Kathuria *et al*., (unpublished) *Histoplasma capsulatum* and histoplasmosis: A review	210
Taphrina deformans	NRRL T-470	Rodrigues and Fonseca, 2003 Molecular systematics of the dimorphic ascomycete genus *Taphrina*. Int. J. Syst. Evol. Microbiol. 53:(PT 2)607-616	216
