# Supplementary material for: RNAseq Analysis Highlights Specific Transcriptome Signatures of Yeast and Mycelial Growth Phases in the Dutch Elm Disease Fungus Ophiostoma novo-ulmi
Source: G3 (Bethesda). 2015 Sep 17;5(11):2487–95. doi: 10.1534/g3.115.021022 (PMC4632067; doi:10.1534/g3.115.021022)
Supplement: Supporting Information [file supp_g3.115.021022_TableS2.pdf]

Table S2 General characteristics for each RNAseq sample for *Ophiostoma novo-ulmi*.

Conditions	Yeast						Solid mycelium						Liquid mycelium					
Replicates	1		2		3		1		2		3		1		2		3	
Duplicates trimmed	No	Yes	No	Yes	No	Yes	No	Yes	No	Yes	No	Yes	No	Yes	No	Yes	No	Yes
#[a] Reads after trimming	1.52E+07	6.81E+06	1.14E+07	5.49E+06	1.21E+07	5.77E+06	1.24E+07	6.31E+06	1.30E+07	6.48E+06	1.71E+07	7.05E+06	1.72E+07	7.92E+06	1.44E+07	6.97E+06	1.36E+07	6.46E+06
# Mapped reads	1.01E+07	4.53E+06	7.59E+06	3.69E+06	7.67E+06	3.79E+06	7.37E+06	4.13E+06	7.92E+06	4.26E+06	9.46E+06	4.22E+06	1.08E+07	5.23E+06	8.92E+06	4.55E+06	8.70E+06	4.28E+06
# Mapped bases	9.87E+08	4.30E+08	7.42E+08	3.51E+08	7.51E+08	3.62E+08	7.21E+08	3.96E+08	7.75E+08	4.07E+08	9.25E+08	3.99E+08	1.06E+09	4.98E+08	8.72E+08	4.33E+08	8.51E+08	4.08E+08
Mean read length	97.87	94.98	97.81	95.21	97.81	95.32	97.82	95.80	97.81	95.63	97.77	94.52	97.82	95.20	97.75	95.30	97.83	95.31
Exons coverage depth	66.26	28.91	49.84	23.60	50.41	24.29	48.43	26.56	52.04	27.34	62.12	26.78	70.93	33.44	58.55	29.10	57.16	27.42
Genome coverage depth	30.04	13.10	22.59	10.70	22.85	11.01	21.96	12.04	23.59	12.40	28.16	12.14	32.16	15.16	26.54	13.19	25.91	12.43
EdgeR normalization factor	0.68	0.84	0.71	0.82	0.85	0.94	1.25	1.15	1.13	1.09	1.28	1.12	1.03	1.04	1.07	1.07	0.90	0.97
# Genes with at least 1 read	8175	8296	8144	8257	8143	8241	8194	8316	8199	8318	8200	8322	8200	8325	8189	8304	8175	8287

[a] #=number
