# Supplementary material for: RNAseq Analysis Highlights Specific Transcriptome Signatures of Yeast and Mycelial Growth Phases in the Dutch Elm Disease Fungus Ophiostoma novo-ulmi
Source: G3 (Bethesda). 2015 Sep 17;5(11):2487–95. doi: 10.1534/g3.115.021022 (PMC4632067; doi:10.1534/g3.115.021022)
Supplement: Supporting Information [file supp_g3.115.021022_TableS3.pdf]

Table S3 Number of orthologous genes found with the reciprocal best blast hits (RBH) method compared with the Inparanoid method of Khoshraftar *et al*. (2013). The number of genes in each species is indicated between parentheses.

Species compared (no of genes)	RBH method	Khoshraftar *et al.* 2013
Ophiostoma ulmi (8639)/ *Neurospora crassa* (9730)	6276	5517
O. ulmi/ Saccharomyces cerevisiae (6604)	3179	2483
O. ulmi/ O. novo-ulmi (8640)	8220	NA[a]
O. novo-ulmi/ N. crassa	6360	NA
O. novo-ulmi/ S. cerevisiae	3260	NA
O. novo-ulmi/ Sporothrix schenckii (8674)	7171	NA
O. novo-ulmi/ Candida albicans (6218)	2774	NA
O. novo-ulmi/ Histoplasma capsulatum (9233)	5292	NA
H. capsulatum / *C. albicans*	3391	NA

[a] NA : data Not Available
