# Supplementary material for: RNAseq Analysis Highlights Specific Transcriptome Signatures of Yeast and Mycelial Growth Phases in the Dutch Elm Disease Fungus Ophiostoma novo-ulmi
Source: G3 (Bethesda). 2015 Sep 17;5(11):2487–95. doi: 10.1534/g3.115.021022 (PMC4632067; doi:10.1534/g3.115.021022)
Supplement: Supporting Information [file supp_g3.115.021022_TableS9.pdf]

Table S9 Description of the 21 orthologous genes overexpressed in yeast in both *Ophiostoma novo-ulmi* and *Candida albicans*. LogFC: average log of fold change between yeast and mycelium phases. FDR ≤ 0.01.

Growth phase	*O. novo-ulmi* genes	Over-expression (logFC)	Description	Candida orthologs	Over-expression (logFC *in C. albicans*)
Yeast	OphioH327g3645	2.76	Glutamate_dehydrogenase	C4_06120W_B	1.38
	OphioH327g4970	3.7	NOL1_NOP2_SUN_domain_containing_protein	CR_02030C_A	0.63
	OphioH327g3717	3.665	Aquaporin-1	CR_02920C_B	1.48
	OphioH327g2293	3.225	Denitrification_regulatory_protein_nirq	C4_00970C_B	0.68
	OphioH327g4808	2.845	Cytosine_deaminase	C6_00620W_B	0.78
	OphioH327g0568	1.93	ATP-dependent_RNA_helicase_ded1	C3_06100C_A	0.71
	OphioH327g2187	1.985	Flocculation_suppression_protein_(Protein_SFL1)	CR_05990C_B	1.15
	OphioH327g2220	2.175	RNA-binding_protein	C5_00790C_A	0.80
	OphioH327g8083	1.76	Low-temperature_viability_protein_ltv1	CR_10650W_A	0.71
	OphioH327g5596	1.71	U3_small_nucleolar_RNA-associated_protein_sof1	C3_00560C_A	0.60
	OphioH327g5986	2.04	Serine/threonine-protein_kinase_srk1_(Sty1-regulated_kinase_1)	C2_07130C_A	0.57
	OphioH327g5054	2.34	Hsp70/Hsp90_co-chaperone_CNS1_(Cyclophilin_seven_suppressor_1/STI1_stress-inducible_protein_homolog)	C1_00560W_A	0.66
	OphioH327g3374	2.28	Fluconazole_resistance_protein_1	C3_06850W_A	1.89
	OphioH327g5556	1.565	DNA-directed_RNA_polymerase,_mitochondrial	C1_00640C_B	0.64
	OphioH327g4855	2.365	U3_small_nucleolar_RNA-associated_protein_20_(U3_snoRNA-associated_protein_20)	C3_01200W_B	0.89
	OphioH327g7825	1.43	RNA_recognition_domain-containing_protein	C3_05150W_B	0.73
	OphioH327g0412	1.255	Fimbrin_(ABP67)	C6_02730W_A	0.50
	OphioH327g7377	2.235	Major_facilitator_superfamily_transporter_multidrug_resistance	C6_04610C_A	0.87
	OphioH327g8261	1.65	Integral_membrane_protein	C1_10360C_A	1.17
	OphioH327g1355	1.38	U3_small_nucleolar_ribonucleoprotein_protein_mpp10	C2_00070C_B	0.60
	OphioH327g5712	1.22	P-type_ATPase	C2_02490C_B	0.76
Mycelium	OphioH327g6748	3.285	N-acetyltransferase-like_protein	C6_00140C_A	0.94
	OphioH327g3302	2.48	IMP-specific_5'-nucleotidase_1	C1_01650W_B	0.72
	OphioH327g0724	2.25	Putative_uncharacterized_protein	CR_09930W_B	0.71
	OphioH327g3285	2.12	Protein_mannosyltransferase_1	C7_02890C_B	0.67
	OphioH327g8280	1.95	UDP-glucose:glycoprotein_glucosyltransferase	C3_02960C_B	0.85
	OphioH327g8395	1.785	Mitogen-activated_protein_kinase	C4_06480C_B	2.05
	OphioH327g4472	1.76	Aromatic_aminotransferase	C2_00340C_A	0.75

OphioH327g5097	1.7	Dolichol-phosphate_mannosyltransferase	C1_08010W_B	0.75
OphioH327g8526	1.655	Rho-GDP_dissociation_inhibitor	C3_05000W_A	1.00
OphioH327g4520	1.75	Ornithine_carbamoyltransferase-like_protein	C6_03230W_B	1.17
OphioH327g6717	1.58	Succinyl-3-ketoacid-coenzyme_a_transferase	C2_07240C_B	1.21
OphioH327g6465	1.61	Fungal_specific_transcription_factor	CR_09210W_A	1.50
OphioH327g2358	1.595	Oligosaccharyl_transferase_subunit	C2_01670C_B	0.74
OphioH327g0333	1.3	Crotonase	C1_03320C_B	0.71
OphioH327g2998	2.545	Aspartic-type_endopeptidase	CR_07800W_B	1.48
OphioH327g4450	1.425	Mannosyltransferase_pmti	C2_06100W_B	0.98
